# Biodegradable, Wear-Resistant and Resilient Thermoplastic Polycarbonate-Based Polyurethane with Nanoscale Microphase Structure

**DOI:** 10.3390/polym17121665

**Published:** 2025-06-16

**Authors:** Shuang Su, Jintao Wang, Qi Yan, Anqi Li, Chuang Liu, Xianli Wu, Yuezhong Meng

**Affiliations:** 1 College of Chemistry, Zhengzhou University, Zhengzhou 450001, China; sushuang365@163.com (S.S.); anqili@gs.zzu.zdu.cn (A.L.); liuchuang@gs.zzu.edu.cn (C.L.); 2 School of Materials Science and Engineering, Zhengzhou University, Zhengzhou 450001, China; jtwang@zzu.edu.cn; 3 Institute of Chemistry, Henan Academy of Sciences, Zhengzhou 450000, China; 4 The Key Laboratory of Low-Carbon Chemistry & Energy Conservation of Guangdong Province, State Key Laboratory of Optoelectronic Materials and Technologies, School of Materials Science and Engineering, Sun Yat-Sen University, Guangzhou 510275, China

**Keywords:** CO_2_-based polycarbonate diol, thermoplastic polyurethane, wear resistance, high resilience, biodegradable

## Abstract

A series of PPCDL-PEG_1000_-TPU were prepared by melting method using CO_2_ based biodegradable polycarbonate diol (PPCDL) and polyethylene glycol (PEG1000) as soft segments, and hexamethylene diisocyanate (HDI) and 1,4-butanediol (BDO) as hard segments. Their structure and properties were characterized to show that the products have nanoscale microphase separation, excellent wear-resistance and high resilience. PPCDL-PEG_1000_-TPUs have high tensile strength, high elongation at break, controllable hardness and excellent wear resistance when the content of hard segment is about 20%. Compared to PPCDL-TPU with only PPCDL as soft segment, the mechanical properties of TPU increase rather than decrease after the addition of PEG due to the crystallization behavior of PEG units in block copolymers. When the ratios of n_PPCDL_:n_PEG_ are 10:1 and 4:1, the tensile strength of PPCDL-PEG_1000_-TPU reaches 27.5 MPa and 16.5 MPa (an increase of nearly 200% and 20% than PPCDL-TPU). The elongation at break reaches 1995% and 2485% (an increase of nearly 40% and 75% than PPCDL-TPU). Hardness of the prepared PPCDL-PEG_1000_-TPUs’ Shore A can be controlled in range of 70~85 by regulating the addition of PEG and their glass transition temperature (Tg) decreases with the increase of the amount of PEG incorporated. All PPCDL-PEG_1000_-TPUs exhibit good wear resistance with the average Akron wear volume of 12 mm^3^ after 4000 cycles of experiments according to national standards. PPCDL-PEG_1000_-TPUs show a high resilience performance with a negligible change in the hysteresis loop area after six cycles of tensile stretching. Furthermore, all PPCDL-PEG_1000_-TPUs possess high thermal stability, strong hydrophobicity, and low water absorption. This material has excellent application prospects and competitiveness in footwear and shock-absorbing materials.

## 1. Introduction

Thermoplastic polyurethane (TPU) is a class of segmented linear polymers containing urethane groups (–NHCOO–) with minimal to no chemical cross-linking. These materials are thermally meltable and can be dissolved in common organic solvents, such as ethyl acetate (EA), chloroform (CHCl_3_), methylene chloride (CH_2_Cl_2_), and tetrahydrofuran (THF) [1]. TPU is primarily composed of two distinct segments: hard segments formed by diisocyanates and chain extenders, and soft segments derived from polyether or polyester polyols. The incompatibility of hard and soft segments in thermodynamics due to a microphase separation structure endows TPU with excellent performance [2]. This structural feature grants TPUs exceptional resistance to hydrolysis, chemicals, abrasion, and impact, coupled with outstanding tensile strength and elasticity [3,4]. TPU has revolutionized industries spanning automotive, wire and cable, medical technology, footwear manufacturing, electronic devices, and beyond, delivering immense value to socio-economic advancement [5,6,7,8,9,10]. The extensive production and consumption of non-biodegradable polyurethanes has brought severe environmental degradation, depletion of non-renewable resources, daunting recycling challenges to the world.

CO_2_, a greenhouse gas, is an industrial byproduct in a great quantity, which can cause global warming. Harnessing chemical carbon capture technologies to transform CO_2_ into valuable, utilizable resources is imperative for environmental protection, combating climate change, and advancing global energy conservation and emission reduction efforts [11,12]. Inspired by triethylboron (TEB)/quaternary ammonium salt catalysts, Meng et al. [13] pioneered a novel metal-free Lewis acid-base catalytic system, ingeniously leveraging 1,4-butanediol as a proton exchange agent to successfully catalyze the copolymerization of propylene oxide (PO) and CO_2_ to synthesize carbon-chemically fixed, fully biodegradable polycarbonate polyol (PPCDL) with precisely controllable molecular weight. This breakthrough is a milestone in sustainable polymer design and preparation because the synthesis of PPCDL harnesses substantial quantities of greenhouse gas CO_2_ by converting it into high-value resources while propelling progress toward the “dual carbon” strategic objectives. TPUs with PPCDL as soft segment has garnered significant interest owing to its cost-effectiveness, minimal environmental impact, exceptional biodegradability, and superior physical properties. They [13] also prepared PPCDL-TPU and PPG-TPU (here, polypropylene glycol (PPG) refers to PPG1000) using the polycarbonate diol as soft segment by the prepolymer method in the solvent under the same conditions to show that PPCDL-TPU has higher hardness, tensile strength, and ultimate elongation than PPG-TPU under the same hard segment conditions, which demonstrates the feasibility of PPCDL as a soft segment in the synthesis of polyurethanes in the polyurethane industry. It is worth pointing out that PPCDL is a fully biodegradable polycarbonate diol, so the PPCDL-TPU possesses certain biodegradability. Huang et al. [14] also prepared PPCDL-PPG-TPU using PPCDL and PPG as mixed soft segments through the solution method. PPCDL-PPG-TPU exhibits good biocompatibility, shape memory effect and excellent biodegradability in both enzyme degradation and compost degradation experiments. Yin et al. [15] synthesized poly(carbonate urethane)s (PCUs) with high transparency and excellent mechanical properties by copolymerization of PPCDL, 4,4-dicyclohexylmethane diisocyanate (HMDI) and 1,4-Butanediol (BDO). The large-scale production of PPCDL has emancipated us from reliance on traditional petrochemical-derived polyols, marking a pivotal milestone in environmental preservation and propelling the sustainable advancement of biodegradable polyurethane materials.

Shoes are essential items for everyone, occupying an important position in our daily life. Natural rubber has been used as shoes’ materials for many years. However, the structure of natural rubber is extremely stable and not easily biodegradable in nature, so the treatment and recycling of waste rubber has remained a significant challenge for the entire rubber industry. The massive accumulation of waste rubber, which stubbornly resists natural biodegradation, has led to a more insidious environmental crisis than plastic’s “white pollution”—a creeping ecological scourge now dubbed “black pollution” [16,17]. Polyvinyl chloride (PVC) is one of the earliest man-made polymer materials to enter the footwear material field. Due to its poor comfort, poor wear resistance, and non-degradability, it is gradually exiting the market under increasing pressure from ecological protection [18,19]. Synthetic rubber [20], thermoplastic rubber (TPR) [21], TPU [22], ethylene vinyl acetate copolymer [23], nylon elastomer [24], and others are also used as shoe materials. Wherein TPU is an ideal choice for shoe materials due to its excellent abrasion resistance, flexibility, low density, light weight, and good physical properties, and has been widely used in the production of shoe materials [25,26,27]. Brückner et al. [28] conducted short-term and long-term mechanical tests on running shoes with polyurethane (PU) and ethylene-vinyl acetate copolymer (EVA) foam soles using hydraulic impact tests. During long-term testing, the damping parameters of EVA foam change more than those of PU materials, confirming the durability and aging resistance of PU. With the innovation of TPU sole materials, not only has the demand for comfort in daily shoes been met, but it has also promoted sustainable development in the TPU footwear manufacturing industry in our country. At present, petroleum resources are becoming increasingly scarce, recycling and utilization costs are high, and they cannot be biodegraded. Seeking green, biodegradable, and renewable materials to replace petrochemical materials has become an inevitable trend in the development of TPU.

Our research group has successfully produced PPCDL with controllable molecular weight and fully biodegradable properties using CO_2_ as one main monomer in large scale. Through the composting experiment testing, PPCDL-TPU and PPCDL-PPG-TPU exhibits excellent biodegradability in both enzyme degradation and compost degradation experiments [14]. Herein, to evaluate the potential of PPCDL-TPU as shoe material, a series of PPCDL-TPUs and PPCDL-PEG-TPUs have been designed and synthesized using PPCDL/PPCDL&PEG as soft segments in this work. The physical properties, thermal properties, water absorption, and wear resistance of the prepared samples were systematically studied to assess their applicability as shoe material. At last, the TPU materials that can meet the requirements of shoe materials have been screened out. This work will champion the widespread integration of eco-friendly PPCDL-TPU materials into everyday life, and drive forward the sustainable development of degradable polyurethane innovations.

## 2. Materials and Methods

### 2.1. Materials

Polycarbonate diol (PPCDL Mn 2000, Foshan Zhongtian Rong New Material Technology Co, Ltd. (Foshan, China)), 1,4-butanediol (BDO, 99%), hexamethylene diisocyanate (HDI, 99%), and dibutyltin dilaurate (for analysis) Dibutyltin dilaurate are from MACKLIN Reagent Company (Shanghai, China). Polyethylene glycol (PEG600, PEG1000, PEG2000) is from Aladdin Reagent Company (Shanghai, China), MACKLIN Reagent Company.

Before being used, PPCDL was needed to be purified and dried to ensure the water content is below 200 ppm. And trace amounts of moisture in the chain extender (1,4-BDO) was needed to remove.

### 2.2. Synthesis of PPCDL-TPU and PPCDL-PEG-TPU

PPCDL-TPU and PPCDL-PEG-TPU were synthesized by solvent-free melt one-step method. First, the PPCDL or/and PEG were added in a three-necked flask and heated to 110 °C in oil bath, vacuum treatment for 2 h, then the reaction system was cooled to 80 °C, and preheated HDI and chain extender (1.4-BDO) were added into the flask and stirred thoroughly for 5 min. The catalyst, dibutyl tin dilaurate, was added into the flask and heated to 160 °C, the reaction was stopped after the appearance of Rod-climbing. The products were removed while hot, aged at 80 °C for 48 h, then cooled down and pressed into a sample strip for characterization. To prevent the TPU from yellowing during the preparation process, the entire preparation process is carried out in a flowing nitrogen atmosphere. Reaction condition screening experiments and results were shown in Table S1.

### 2.3. Measurement Methods

The structures of the polyether polyols and the synthesized TPU was studied using a Fourier transform infrared spectrometer (FT-IR-100, PerkinElmer, Waltham, MA, USA) and Oxford NMR spectrometer (Quantum-I Plus 400 MHz, Wuhan, China). The TPU powder was ground and mixed with potassium bromide using the potassium bromide pellet method for FTIR tests. Test conditions are scanning wavenumber range of 4000–400 cm^−1^ and resolution of 4 cm^−1^. H^1^-NMR spectrum was detected by solving 5 mg PPCDL or TPU samples in deuterated chloroform with tetramethylsilane as the internal standard. The Mn, Mw, and PDI of TPU were tested by GPC produced by Waters Corporation of the United States, with polystyrene as the standard and tetrahydrofuran (THF) as the mobile phase. Before using GPC to determine the molecular weight of samples, we calibrate the GPC instrument using standard substances to obtain an effective standard curve with an R^2^ of 0.9992 and an R of 0.99991. The glass transition temperature (Tg) of PPCDL and the prepared TPUs was tested using a differential scanning calorimeter (DSC) from NETZSCH (Selb, Germany). The test temperatures were set at −20 °C~120 °C for the first stage and −40 °C~145 °C for the second stage, with a heating rate of 10 K/min. The Tg values were read from the second stage. Atomic force microscopy (AFM) was conducted on a Bruker Dimension Fastscan in peak force tapping and quantitative nano-mechanical mapping mode. The atomic force microscopy images were processed using Gwyddion-2.52 (https://gwyddion.net/) and SPM-9700 software (https://www.shimadzu.com.cn/). The fracture surface of specimens treated by liquid nitrogen was used for testing. Thermogravimetric analysis test was conducted on the sample using a Labsys thermogravimetric analyzer produced by Setaram Instrumentation (Caluire-et-Cuire, France). The test temperature was divided into two stages: the first stage involves heating up to 120 °C and holding for 300 s, and the second stage involves heating from 120 °C up to 800 °C at a rate of 10 K/min. Tensile property testing was conducted in accordance with the standard GB/T 528-2009 using the TH-8203A desktop tensile machine produced by Suzhou Top Instrument Co., Ltd. (Suzhou, China). Five Type 2 dumbbell specimens prepared according to GB/T 528-2009 were selected for testing. The value with the largest deviation was removed, and the median value from the results was chosen as the experimental outcome. The hardness test is conducted in accordance with GB/T 531.1-2008, selecting samples with a thickness greater than 6 mm for testing with a Type A durometer from Suzhou Test Instrument Co., Ltd. (Suzhou, China). The hardness at five different locations is tested, and the results are averaged. The CSI-A00 Akron abrasion tester produced by Chengwei Instruments (Tianjin, China) is used to conduct wear resistance tests on four groups of TPU and calculate the abrasion.

## 3. Results and Discussion

### 3.1. The Structure of PPCDL-TPU and PPCDL-PEG_1000_-TPU

**Figure 1:**
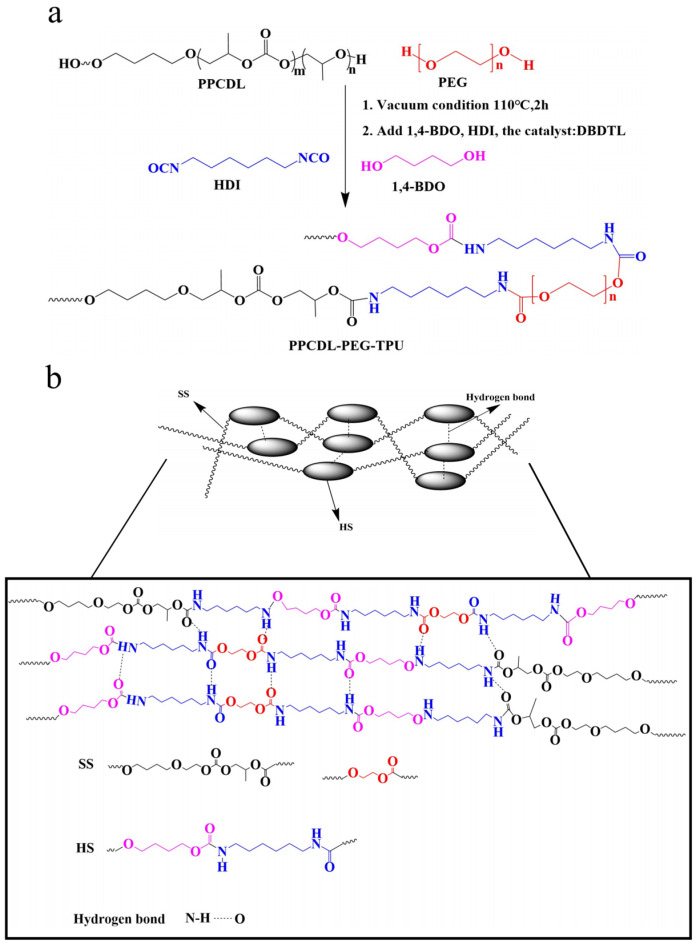
(**a**) The synthesis process and (**b**) schematic diagram of TPUs architecture with the mixture of PPCDL and PEG as soft segments and HDI and 1,4-BDO as hard segments.

To determine the effect of soft segment types and contents, hard segment, and R (n_NCO_/n_OH_) values on the structure and properties of the prepared TPUs, a series of comparative tests (as shown in Tables S1–S4) were conducted. By preliminary screening, an R value of 1.05 and 20% hard segment content were chosen to study the effect of incorporating different proportions of PEG on the performance of PPCDL-TPU, using PPCDL, PEG1000 (here 1000 refers to the Mn of PEG), HDI, and 1,4-BDO as raw materials. Figure 1a presents a detailed schematic diagram of the reaction pathways involved in the preparation of TPUs, emphasizing the critical steps necessary for successful TPU synthesis. Figure 1b shows the microstructure of the prepared TPU. The mole ratios of the PPCDL and PEG1000 were set up as 4:1, 6:1, 8:1, 10:1, and the corresponding products named as TPU4, TPU6, TPU8, TPU10 respectively. The product without PEG1000 was recorded as TPU0. The formulation and molecular weight of the TPUs are shown in Table 1. The molecular weight of all TPUs was determined using GPC with THF as the mobile phase, and the GPC curves are presented in Figure S1. All of the prepared TPUs have a number-average molecular weight of over 36,000 Da, meeting the molecular weight requirements for thermoplastic polyurethane elastomers [29]. With the addition of PEG1000, there is a significant increase in the molecular weight. The PDI of all TPUs is around 1.5, indicating the feasibility of preparing PPCDL-TPU using a solvent-free melt one-step method.

The ^1^H-NMR spectra and structural formulas of TPU0, TPU10 and PPCDL were shown in Figure S3. In the PPCDL spectrum, the two relatively strong signals at 5.0 ppm and 4.3–4.0 ppm correspond to the signal peaks of –CH– and –CH_2_– in the polycarbonate segment. The signals at 3.3–3.6 ppm and 1.14 ppm are attributed to the signal peaks of –CH– and –CH_2_– connected by ether bonds, with the methylene absorption peaks in –OCH_2_CH_2_CH_2_CH_2_O– found at 3.40 ppm and 1.8 ppm. Upon the polymerization of PPCDL with HDI and BDO, the grafting success can be observed from the 1H-NMR spectrum, where the –CH_2_– shift directly connected to the urethane group on HDI appears at 3.3 ppm. The peak at 3.5 ppm belongs partly to the “H” in PPCDL and partly to the “H” connected to the oxygen atom in BDO. After adding PEG1000, there is a strong absorption peak at 3.7 ppm, attributed to the two equivalent hydrogens (i) directly connected to the urethane groups formed by PEG and HDI. This indicates that PPCDL-TPU and PPCDL-PEG_1000_-TPU have been successfully synthesized.

**Figure 2:**
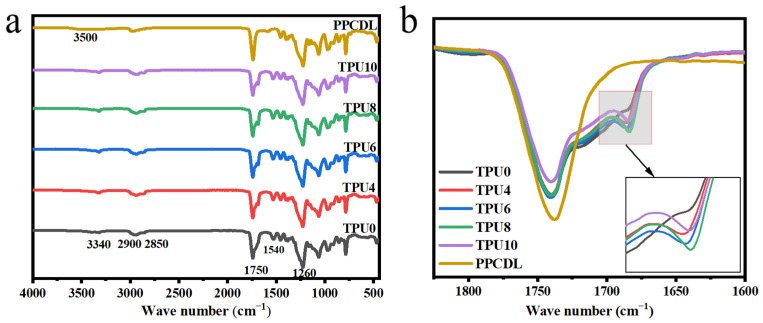
FT-IR spectra of (**a**) PPCDL and the prepared TPUs, (**b**) zoomed-in view from 1600 cm^−1^ to 1900 cm^−1^ of (**a**).

FTIR spectra of PPCDL and the prepared TPUs are shown in Figure 2a. There are strong stretching vibration absorption bands at 1750 cm^−1^ and 1260 cm^−1^ in the FTIR spectrum of PPCDL, attributed to the carbonyl group in the carbonate moiety (O–C=O) and the ether bond. The strong stretching vibration absorption peak of the hydroxyl group (–OH) characteristic of PPCDL appears at 3500 cm^−1^. The stretching vibration absorption bands at 2900 cm^−1^ and 2875 cm^−1^ belong to the –CH_3_ and –CH_2_– groups. The characteristic absorption peak at 3500 cm^−1^ disappears in FTIR spectra of TPUs. New peaks appear at 3340 cm^−1^ and 1540 cm^−1^, which are attributed to the stretching vibration and in-plane bending vibration of the N–H bond. Furthermore, compared with PPCDL, the enhanced absorption of stretching vibration at 1750 cm^−1^ indicates that the polyurethane groups have successfully formed, resulting in a large number of absorption peaks for carbonyl groups. As can be seen in Figure 2b, TPU0, TPU4, TPU6, TPU8, and TPU10 all exhibit shoulder peaks at the carbonyl peak of 1680~1760 cm^−1^. The absorption peak at 1750 cm^−1^ corresponds to the free carbonyl group, and the absorption peak at 1680 cm^−1^ is corresponding to the hydrogen-bonded carbonyl group because hydrogen bonds can cause the stretching vibration of the carbonyl group to shift towards lower wavenumbers [30], manifesting a large number of hydrogen bonds formed between the soft and hard segments. The characteristic absorption peak of –NCO does not exist at 2270 cm^−1^, indicating that the –NCO has completely reacted with the –OH in the system [31]. In summary, all TPUs have been successfully prepared.

**Figure 3:**
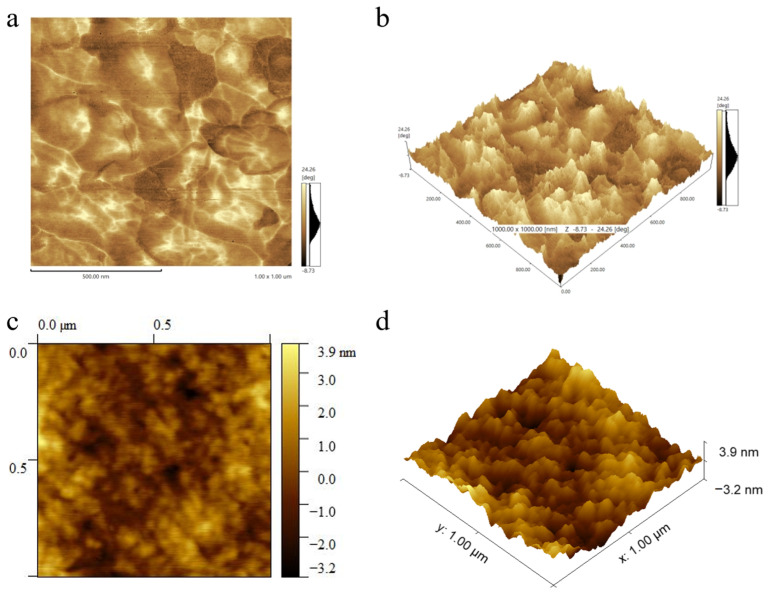
AFM graphs of (**a**) TPU10 and (**c**) TPU8; 3D Height Retrace of (**b**) TPU10 and(**d**) TPU8.

AFM was used to confirm the microfacies distribution state. The graphs of AFM, as shown in Figure 3, indicate that PPCDL-PEG_1000_-TPU samples have relatively uniform nanoscale phase separation. The bright areas correspond to the hard segments of the TPU with a diameter of about 85 nm, which are evenly distributed, while the dark areas represent the distribution regions of the soft segments [32,33]. There is no distinct interface between the soft and hard segments, exhibiting a distribution state of semi-network and semi-domain areas. The soft segments provide excellent tensile properties for TPU, while the hard segments form microphase-separated structures of certain dimensions through hydrogen bonding for physical crosslinking, which can disperse and transmit external forces, effectively enhancing the physical properties of TPU [34,35].

### 3.2. Properties of the Synthesized TPUs

#### 3.2.1. Thermal Properties

**Figure 4:**
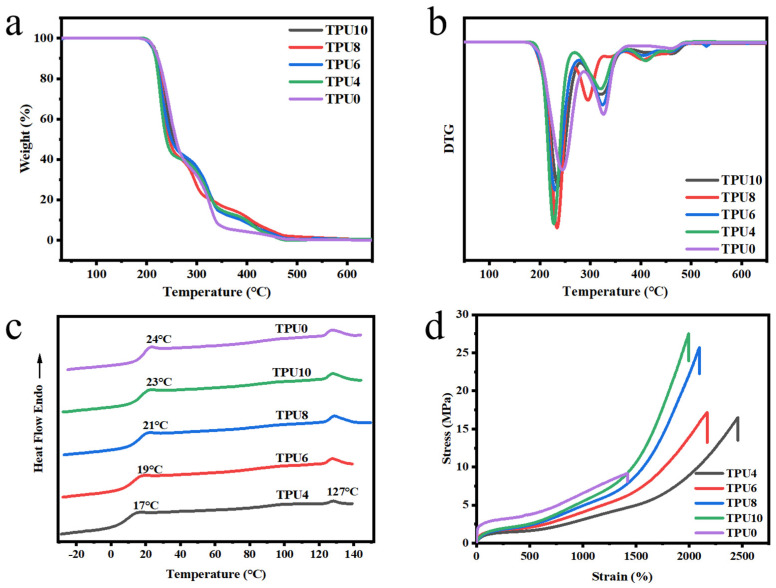
(**a**) TGA curves and (**b**) weight-loss derivative curves of TPUs; (**c**) DSC curves of PPCDL and the prepared TPUs (the second heating run); (**d**) Tensile stress-strain curves of TPUs.

The TGA and DTG curves of the prepared TPUs are shown in Figure 4a,b. Pyrolysis temperature ranges are divided based on the coordinate values corresponding to the decomposition peaks in DTG combined with the number of weight loss steps in the TGA curve. It is evident that the temperature of all TPUs at a weight loss of T_5%_ is around 210 °C, indicating that the prepared TPUs possess good thermal stability [36]. The DTG curves of TPUs exhibits two distinct weight loss peaks in ranges of 210 °C~250 °C and 280 °C~350 °C. the first thermogravimetric peak is attributed to the decomposition of the carbonate groups in segment of PPCDL. The maximum weight loss occurs at the first step due to the soft segment content of TPU up to 80%. The second stage of thermal decomposition in the range of 280 °C~350 °C was corresponded to the decomposition of urethane groups in the hard segments. Compared to TPU0, TPUs with PEG1000 and PPCDL as mixed soft segments exhibit a new thermogravimetric peak at 380 °C~420 °C, which is attributed to the decomposition of the polyether PEG1000. It is evident that all TPUs possess good thermal stability.

Tg of TPU needs to be comprehensively designed according to specific application scenarios, with priority given to selecting TPU with low Tg for flexible and low-temperature requirement scenarios. As shown in Figure 4c, all TPUs exhibit a transition in ranges of 17–23 °C corresponding to Tg of soft segment and a melting peak at near 127 °C belonging to melting of hard segment, indicating that the prepared TPUs possess favorable microphase separation. The prepared PPCDL-PEG_1000_-TPUs show lower Tg than TPU0 because PEG1000 is a linear long-chain polyether. The incorporation of PEG into the soft segments can significantly reduce the Tg of TPUs. When the ratio of n_PPCDL_:n_PEG_ reduces from 10:1 to 4:1, the Tg drops from 23 °C to 17 °C, mainly attributed to the fact that the polyether PEG1000 endows the TPU molecular chains with better flexibility, enhancing the mobility of the molecular chains [37].

#### 3.2.2. Mechanical Properties

Tensile properties are the most fundamental properties of elastomers, and TPU with greater tensile strength and elongation at break can be applied in a more diverse range of scenarios. Compared to TPU0, TPUs with the same hard segment content exhibit higher tensile strength and better elongation at break after introducing PEG1000 into the soft segment, shown as Figure 4d. TPU0 exhibits a high hardness (Shore A 90 ± 1) and a low tensile strength (8.9 MPa), shown as Table 2. When the ratios of nPPCDL:nPEG are 10:1 and 4:1, the tensile strength of TPUs reaches 27.5 MPa and 16.5 MPa (an increase of nearly 200% and 20% than TPU0) and the elongation at break reaches 1995% and 2485% (an increase of nearly 40% and 75% than TPU0) due to the presence of more C–O–C single bonds in the molecular structure of polyether PEG, which gives the ether bonds a stronger ability for internal rotation and crystallinity [33]. The crystallization behavior of PEG units in block copolymers is strongly influenced by the nature of adjacent groups interacting with them (e.g., urethane groups), thereby enhancing the level of polymer phase separation, which can increase the tensile strength of TPU [38]. When the strain is below 1500%, it corresponds to the stretching of the soft segment chains, where the soft segment chains (PPCDL and PEG1000) and a small amount of hard segments undergo elastic deformation through chain segment movement. Under high strain, the molecular chains of the soft segments are fully stretched and highly oriented. At this point, due to the strong phase-separated structure within the system, the hard segments continue to transmit stress as rigid microdomains. Since the hard segment consists of linear hexamethylene diisocyanate, its deformability is significantly lower than that of the macromolecular PPCDL and PEG. Therefore, the subsequent deformation of 1500–2000% is provided by the hard segment region. Moreover, the hard segment exhibits a linear structure capable of withstanding greater tensile forces provided by the machine, while its deformation capacity is poor. Macroscopically, this manifests as a significant variation in the material’s tensile strength within the range of 1500~2500% [39,40,41]. At the same time, the PEG segments are relatively softer than PPCDL, which endows the TPU segments with a certain degree of mobility. Therefore, the addition of PEG also facilitates the regulation of the hardness of PPCDL-TPU [42,43,44]. Within the range of n_PPCDL_:n_PEG_ from 10:1 to 4:1, the hardness (Shore A) of TPUs is between 85 ± 1 and 72 ± 1. The prepared materials are relatively soft and very transparent. As shown in Figure S2, all PPCDL-PEG_1000_-TPUs films and strands exhibit good transparency, especially the films, which are difficult to observe due to their excellent transparency. This provides it with a broad application prospect.

**Figure 5:**
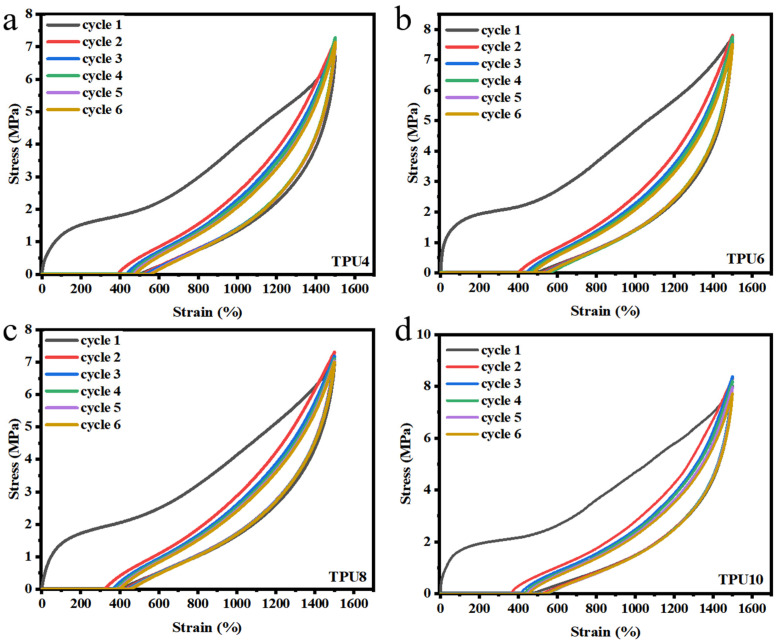
The cyclic tensile curve test (six cycles) of (**a**) TPU4, (**b**) TPU6, (**c**) TPU8, (**d**) TPU10.

Resilience is very important for TPU, and TPUs with higher resilience can replace certain rubber materials for the production of shoe soles. The cyclic tensile curves of PPCDL-PEG_1000_-TPUs demonstrate impressive deformation recovery rate, showing exceptional fatigue resistance, as shown in Figure 5a–d. From its stress-strain curve, it is evident that the permanent deformation produced by mechanical hysteresis after the first loading is relatively large due to the fact that the loading process disrupts the hydrogen bonding forces within the TPU molecular chains, which prevents them from fully recovering [45]. The enclosed area in the stretching-recovery curve for each cycle represents the energy loss during the process. The percentage of enclosed area generated after the second and third cycles of all TPUs significantly decreases, and the permanent deformation also changes slightly. The deformation reduction of TPUs has become slower compared to the previous time, because there are more continuous crystalline regions in the polymer. After the second loading, the non-recoverable crystalline regions decrease, and accordingly, the mechanical hysteresis percentage significantly decreases, and the permanent deformation changes are also smaller [46]. All PPCDL-PEG_1000_-TPUs show a gradually decreasing hysteresis loop area formed by the loading and unloading curves after the second loading, indicating that PPCDL-PEG_1000_-TPUs possess high resilience [47]. At a fixed elongation of 1500%, the tensile strength of PPCDL-PEG_1000_-TPUs is above 7 MPa, which makes them ideal for applications requiring ultra-high resilience. The digital photo of deformation during the tensile process was shown in Figure S4. With the increase in the amount of PEG1000, there is a trend of decreasing tensile strength, which is in accordance with the expected pattern [48]. Furthermore, there is no significant decrease in the tensile strength of the material after being cyclically stretched 6 times, exhibiting excellent resilience.

#### 3.2.3. Hydrophobicity and Water Absorption

**Figure 6:**
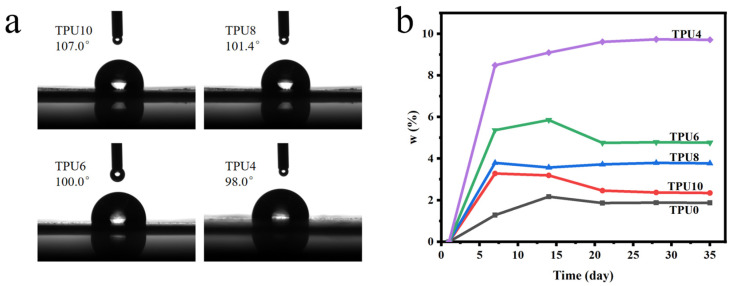
(**a**) Contact angle and (**b**) Water absorption curves of the prepared TPUs.

Due to the large number of carbonate bonds in PPCDL, prolonged contact with water can easily lead to the decomposition of the carbonate groups. Therefore, the contact angle and water absorption were measured to characterize the water resistance of the prepared TPUs. All PPCDL-PEG_1000_-TPUs possess good hydrophobic properties, with measured water contact angles all greater than 90°, shown as Figure 6a. The water absorption rates of TPUs after immersion in water for 35 days are all less than 10%, as shown in Figure 6b. PEG is a highly flexible hydrophilic polymer, hence the water absorption rate increases with the increase of PEG contents [49,50,51].

#### 3.2.4. Akron Abrasion

**Figure 7:**
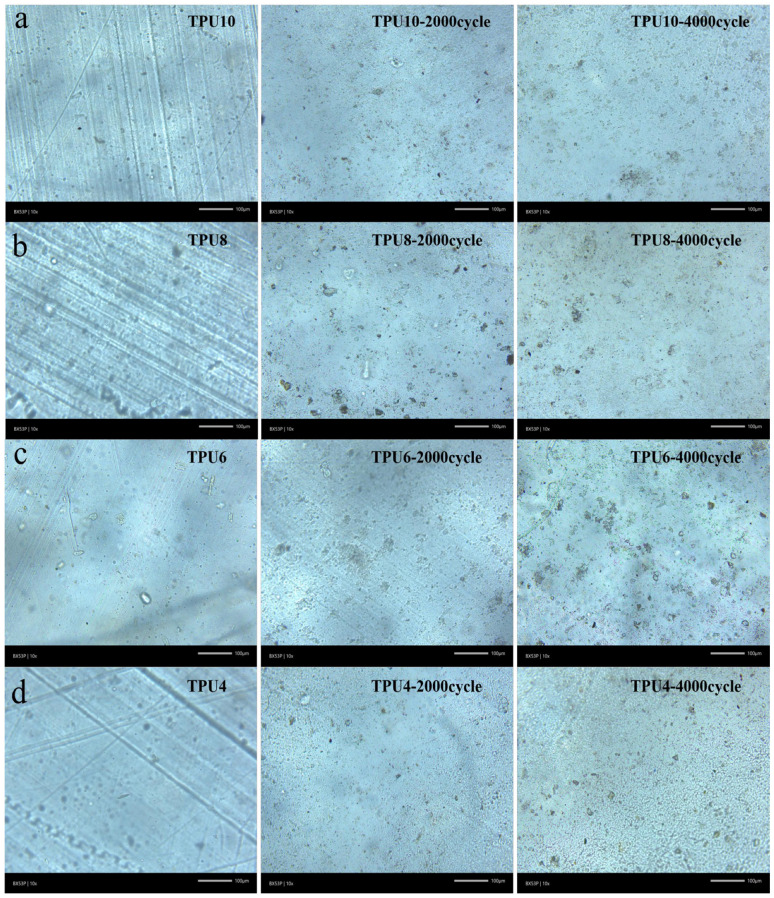
Comparison of (**a**) TPU10 (**b**) TPU8 (**c**) TPU6 (**d**) TPU4’s surface optical microscopy before and after Akron Abrasion experiments.

The wear resistance of the sole, the component that directly contacts the ground, directly determines the service life and safety of the shoe. The details of the wear resistance test process are shown in Figure S5. After being crushed, the PPCDL-PEG_1000_-TPUs were pressed into corresponding strands according to the G/BT1689-2014 standard for abrasion testing, with the results shown in Table 3. After conducting a 2000-cycle test in accordance with national standards, no loss in quality was observed in any of the TPU samples. The experiment was continued up to 4000 cycles, resulting in a slight quality loss in all TPU samples, approximately 0.02 g. As can be seen from Figure 7, the surface roughness of the specimen changed before and after the wear test, and no significant weight variation occurred due to tearing or detachment of the sample. This is primarily attributed to the excellent resilience of all TPUs, which only exhibit surface indentation and minimal weight loss after friction [52,53,54]. This indicates that the prepared PPCDL-PEG_1000_-TPUs possess excellent wear resistance. Moreover, as shown in Figure 4d, as the hardness of samples gradually decreases, the alteration of surface flatness by the friction process becomes particularly pronounced.

## 4. Conclusions

In summary, we achieved the successful synthesis of a novel series of PPCDL-PEG_1000_-TPU through one step melting method by employing CO_2_ based biodegradable polycarbonate diol PPCDL, PEG1000, HDI, and BDO as raw materials. All TPUs possess good transparency. In contrast to PPCDL-TPU, incorporating a precise ratio of PEG1000 into the soft segment enables the creation of PPCDL-PEG_1000_-TPU with controllable hardness, exceptional tensile strength, remarkable elongation at break, and superior wear resistance. This innovative formulation achieves distinct nano-scale microphase separation while maintaining a remarkably low hard segment content of just 20%. All TPUs exhibit excellent thermal stability, high resilience, minimal water absorption, and inherent hydrophobicity. Their performance significantly exceeds the national standards for footwear materials and they have a broad range of application prospects. This promotes the use of biodegradable PPCDL-TPU in daily life and facilitates the green and sustainable development of the polyurethane industry.

## Figures and Tables

**Table 1:** Preparation of TPUs with PPCDL and PEG1000 as soft segments.

Samples	Ingredient				Mn (Da)	PDI
	PPCDL ^a^ (g)	PEG_1000_^b^ (g)	HDI (g)	BDO (g)		
TPU0	20.05	0	3.91	1.09	36,586	1.53
TPU10	20.28	1.01	4.18	1.13	102,089	1.46
TPU8	20.74	1.33	4.35	1.16	94,492	1.50
TPU6	20.03	1.69	4.29	1.13	56,077	1.54
TPU4	20.56	2.60	4.27	1.01	64,498	1.62

a: OH value 56.1 mg/g. b: OH value 112.2 mg/g. n_NCO_/n_OH_ = 1.05, content of hard segment = [(mass of HDI + mass of BDO)/(mass of HDI + mass of BDO + mass of PEG1000 + mass of PPCDL)] × 100%.

**Table 2:** Mechanical properties and hardness of TPUs with different proportions of PEG1000.

	TPU0	TPU10	TPU8	TPU6	TPU4
Hardness (Shore A)	90 ± 1	85 ± 1	83 ± 1	75 ± 1	72 ± 1
σ (MPa)	8.9	27.5	25.6	17.1	16.5
ε (%)	1419	1995	2094	2169	2458
ε 1500% (MPa)	/	8.39	7.28	7.81	7.31

**Table 3:** Wear volume of TPUs.

Sample	TPU10	TPU8	TPU6	TPU4
2000 circle Abrasion (cm^3^)	0	0	0	0
4000 circle Abrasion (cm^3^)	0.0120	0.0123	0.0119	0.0117

## Data Availability

The original contributions presented in this study are included in the article/Supplementary Material. Further inquiries can be directed to the corresponding author.

## Supplementary Materials

The following supporting information can be downloaded at: https://www.mdpi.com/article/10.3390/polym17121665/s1, Figure S1: GPC curves of the prepared TPUs; Figure S2: PPCDL-PEG_1000_-TPUs’ film and profile appearance; Figure S3: (a) ^1^H-NMR spectrum of purified PPCDL, (b) ^1^H-NMR spectrum of TPU0, (c) ^1^H-NMR spectrum of TPU10; Figure S4: Deformation of PPCDL-PEG_1000_-TPU film during tensile process; Figure S5: PPCDL-PEG_1000_-TPU Wear Resistance Test; Table S1: Preparation of PPCDL-TPU with Different R Values; Table S2: Preparation of PPCDL-TPU with Different Hard Segments; Table S3: Preparation of PPCDL-TPU Using Different Isocyanates; Table S4: Preparation of PPCDL-PEG-TPU with different molecular weights of PEG.
