# Supplementary material for: Biodegradable, Wear-Resistant and Resilient Thermoplastic Polycarbonate-Based Polyurethane with Nanoscale Microphase Structure
Source: Polymers (Basel). 2025 Jun 16;17(12):1665. doi: 10.3390/polym17121665 (PMC12197117; doi:10.3390/polym17121665)
Supplement: Supplementary file 1 [file polymers-17-01665-s001.zip › polymers-3619247-supplementary.pdf]

Table of Content **Page**

Table S1. Preparation of PPCDL-TPU with Different R Values S1

Table S2. Preparation of PPCDL-TPU with Different Hard Segments S2

Table S3. Preparation of PPCDL-TPU Using Different Isocyanates S2

Table S4. Preparation of PPCDL-PEG-TPU with different molecular weights of PEG S2

Figure S1. GPC curves of the prepared TPUs S3

Figure S2. PPCDL-PEG_{1000}-TPUs' film and profile appearance S3

Figure S3. (a) ^{1}H-NMR spectrum of purified PPCDL , (b) ^{1}H-NMR spectrum of TPU0, (c) ^{1}H-NMR spectrum of TPU10 S4

Figure S4. Deformation of PPCDL-PEG_{1000}-TPUfilm during tensile process S5

Figure S5. PPCDL-PEG_{1000}-TPU Wear Resistance Test. S5

Table S1. Preparation of PPCDL-TPU with Different R Values

Samples	Diol	Isocyanate	Chain extender	ShoreA/D	Stress(MPa)	Strain(%)	Tg(°C)
R0.95	PPCDL	HMDI	BDO	60	3.7	716	21.0
R1.0	PPCDL	HMDI	BDO	65	4.8	645	27.0
R1.05	PPCDL	HMDI	BDO	95/65	22.3	191	31.9
R1.1	PPCDL	HMDI	BDO	95/70	31.0	50	38.0
R1.15	PPCDL	HMDI	BDO	98/75	42.5	28	39.5
R1.2	PPCDL	HMDI	BDO	98/80	47.0	15	40.0

Table S2. Preparation of PPCDL-TPU with Different Hard Segments

Samples	Diol	Isocyanate	Chain extender	ShoreA/D	Stress(MPa)	Strain(%)	Tg(°C)
TPU15%	PPCDL	HMDI	BDO	65	4.1	725	26.2
TPU20%	PPCDL	HMDI	BDO	95/65	22.3	191	31.9
TPU25%	PPCDL	HMDI	BDO	98/72	28.0	75	40.0
TPU30%	PPCDL	HMDI	BDO	99/74	58.0	25	45.0

As shown in Table S1,S2, As the R value and hard segment content increase, the

tensile strength, hardness, and Tg of PPCDL-TPU also increase. However, when R > 1.05, the prepared TPU with a hardness of Shore A95/Shore D65 is difficult to apply. The increase in hard segment content enhances the degree of microphase separation and the number of hydrogen bonds in TPU, providing more physical cross-linking points, thereby improving the mechanical properties of the material.

Table S3. Preparation of PPCDL-TPU Using Different Isocyanates

Samples	Diol	Isocyanate	Chain extender	ShoreA/D	Stress(MPa)	Strain(%)	Tg(°C)
MDI-TPU	PPCDL	MDI	BDO	99/76	37.0	13	38.5
HMDI-TPU	PPCDL	HMDI	BDO	98/69	24.0	196	39.0
IPDI-TPU	PPCDL	IPDI	BDO	98/60	7.4	593	29.2
HDI-TPU	PPCDL	HDI	BDO	90	9.2	1414	20.9

As shown in Table S3, The hardness, tensile strength, and Tg of PPCDL-TPU are related to the structure of the diisocyanate used as raw material. Typically, aromatic > alicyclic > aliphatic diisocyanates. The more symmetrical the structure of the diisocyanate, the greater the rigidity of the molecular structure, and the smaller the steric hindrance of the —NCO groups, the higher the hardness, tensile strength, and Tg of the resulting TPU.It is interesting to note that HDI-TPU possesses an outstanding elongation at break of up to 1414%, although its tensile strength is lower than that of HMDI-TPU (24.0MPa) and MDI-TPU (43.0MPa). However, it has a moderate hardness (Shore A 90), and its tensile strength and elongation at break can potentially be improved through modification.

Table S4. Preparation of PPCDL-PEG-TPU with different molecular weights of PEG

Samples	Diol	Isocyanate	Chain extender	ShoreA/D	Stress(MPa)	Strain(%)	Tg(°C)
TPU600	PPCDL/PEG600	HDI	BDO	85	4.7	950	16.0
TPU1000	PPCDL/PEG1000	HDI	BDO	83	25.6	2094	23.0
TPU2000	PPCDL/PEG2000	HDI	BDO	80	9.3	1783	16.5

In summary, PPCDL-TPUs prepared using single PPCDL as the soft segment exhibit greater hardness, higher tensile strength, and lower elongation at break. To optimize cost control, all experiments utilized a fixed R-value of 1.05, maintained a hard segment composition of 20%, and employed BDO as the chain extender. As shown in Table S4, When using HDI as a raw material, the prepared PPCDL-TPU has good tensile strength, but the hardness is higher. It is exciting that upon introducing polyether PEG into its soft segments, the addition of PEG1000 not only enhances the elongation at break and tensile strength but also reduces its hardness.

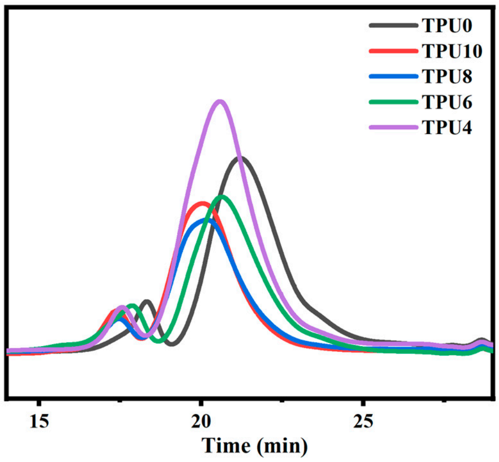

Figure S1. GPC curves of the prepared TPUs

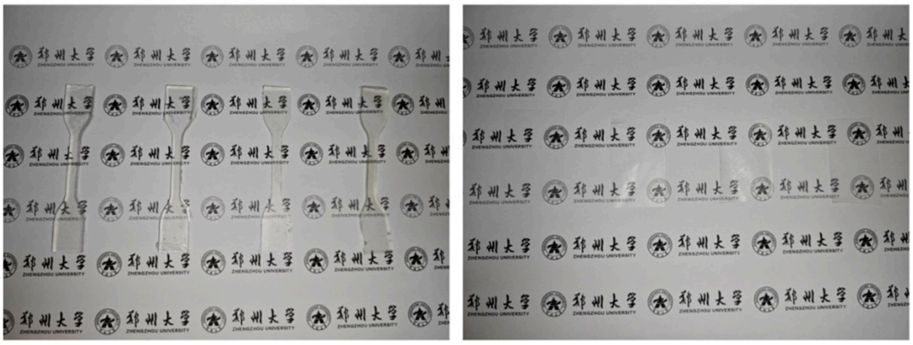

Figure S2. PPCDL-PEG$_{1000}$-TPUs' film and profile appearance

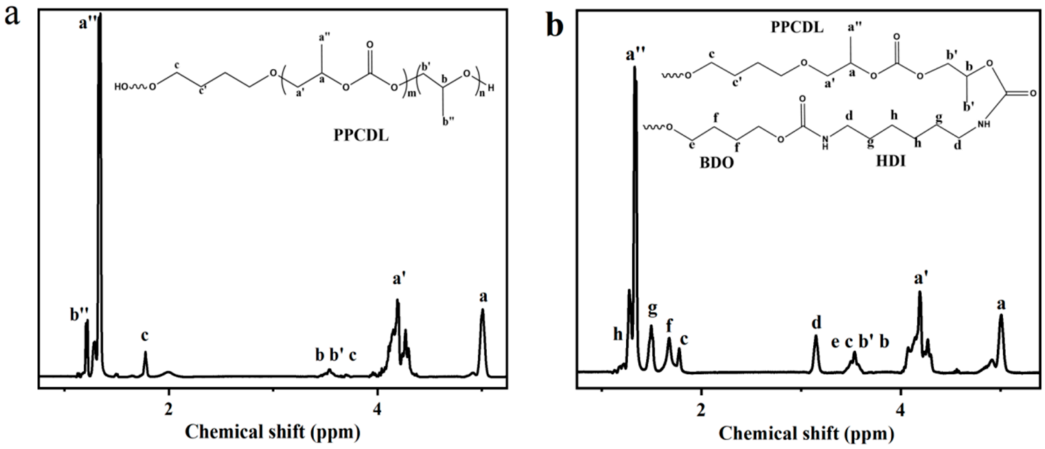

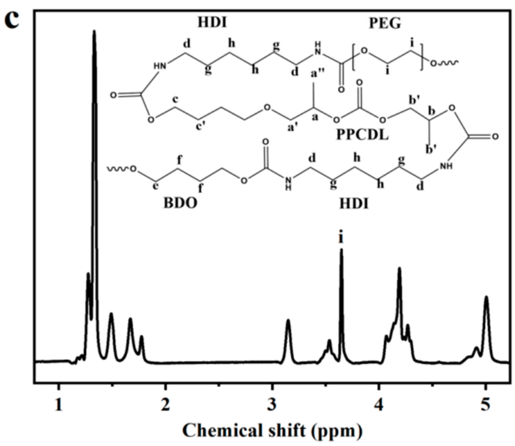

Figure S3. (a) ^{1}H-NMR spectrum of purified PPCDL , (b) ^{1}H-NMR spectrum ofTPU0, (c) ^{1}H-NMR spectrum of TPU10

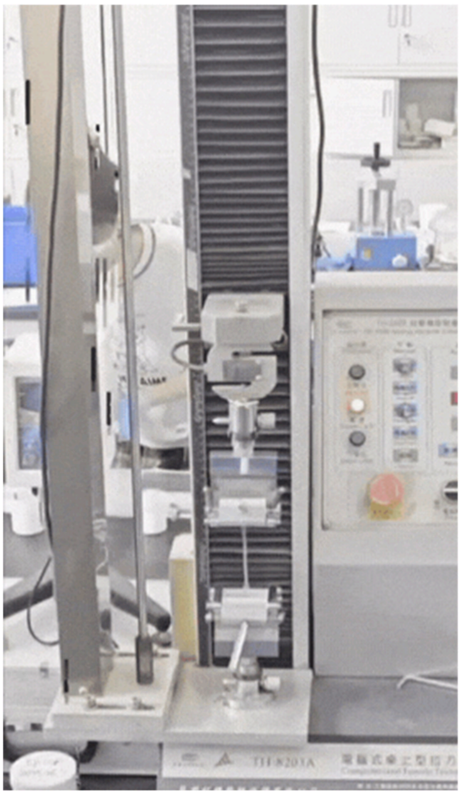

Figure S4. Deformation of PPCDL-PEG$_{1000}$-TPUfilm during tensile process.

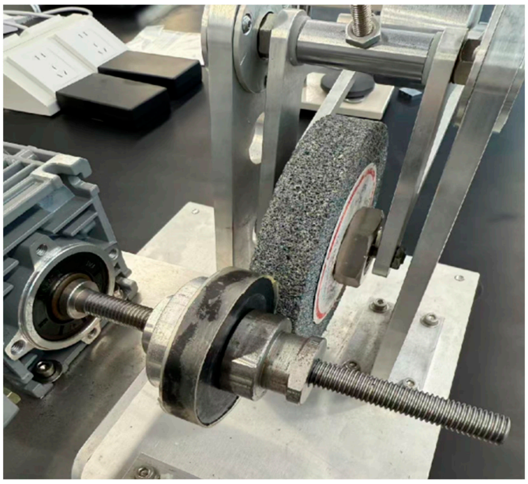

Figure S5. PPCDL-PEG_{1000}-TPU Wear Resistance Test.
